# Potential of mobile technology in meeting the public health needs in developing countries

**DOI:** 10.1186/1471-2458-12-S2-A13

**Published:** 2012-11-27

**Authors:** Isidore Koffi Kouadio, Hasanain Faisal Ghazi, Nimetcan Mehmet Orhun, Azam Rahimi, Syed Mohamed Aljunid

**Affiliations:** 1 United Nations University-International Institute for Global Health, Universiti Kebangsaan Malaysia Medical Centre, Jalan Yaacob Latiff, 56000 Kuala Lumpur, Malaysia; 2 Department of Community Health, Universiti Kebangsaan Malaysia Medical Centre, Jalan Yaacob Latiff, 56000 Kuala Lumpur, Malaysia

## Background

Disease epidemics and shortage of healthcare professionals present major challenges for developing countries in achieving the Millennium Development Goals (MDGs), specifically the health related goals (4, 5 and 6). In these countries, the explosive growth of mobile communications offers a new opportunity for the promotion of quality healthcare. There is a growing body of evidence that demonstrate the potential of mobile communication to further improve awareness and healthcare services, even in some remote and poor-resources environment.

## Objectives

The aim of this paper was to describe the opportunity that represents the mobile technology in developing countries and to summarize its different applications in meeting their public health challenges.

## Materials and methods

A comprehensive review was conducted from January 14^th^ to February 30^th^ 2012 through PubMed, WHO, United Nation Foundation and Google scholar websites using combination of several keywords: Mobile Technology, Mobile Health, Public Health, Developing countries.

Articles and Reports describing the potential of mobile health in developing countries were included in the study.

## Results

Mobile phone reaches further into developing countries than any other information and communication technology (ITC) and health infrastructures. By the end of 2008, the global use of mobile phones had increased to about 4.1 billion. A full 64% of users are found in developing countries. In Africa alone, mobile phone sales have grown by 550 percent in a period of five years. The mobile health fields are remarkably dynamics and the range of applications is constantly expanding. The key applications in meeting the health needs in developing countries are respectively (1) Remote data collection; (2) Remote monitoring; (3) Diagnostic and treatment support; (4) Diseases and epidemic outbreak tracking; (5) Education and awareness; (6) Communication and training for healthcare workers and (7) Disaster warning.

## Conclusions

For sustainability and scalability mobile health programs need to be based on key building blocks and strong partnerships. Scientific program evaluation and further research are needed in the field of mobile health in developing countries.

